# Outpatient administration of BEAM conditioning prior to autologous stem cell transplantation for lymphoma is safe, feasible, and cost‐effective

**DOI:** 10.1002/cam4.879

**Published:** 2016-10-03

**Authors:** Robin M. Reid, Andrea Baran, Jonathan W. Friedberg, Gordon L. Phillips, Jane L. Liesveld, Michael W. Becker, Lucy Wedow, Paul M. Barr, Laurie A. Milner

**Affiliations:** ^1^ James P Wilmot Cancer Institute and Department of MedicineUniversity of Rochester Medical CenterRochesterNew York; ^2^ Rochester Regional HealthRochesterNew York; ^3^ Wake Forest Baptist HealthWinston‐SalemNorth Carolina; ^4^ Department of Pathology and Laboratory MedicineUniversity of Rochester Medical CenterRochesterNew York

**Keywords:** Complications, conditioning chemotherapy, cost, stem cell transplantation

## Abstract

High‐dose BEAM chemotherapy (BCNU, etoposide, Ara‐C, and melphalan) followed by autologous hematopoietic stem cell transplantation is frequently used as consolidative therapy for patients with recurrent or refractory Hodgkin or non‐Hodgkin lymphoma. The BEAM regimen has traditionally been administered over 6 days in the hospital, with patients remaining hospitalized until hematologic recovery and clinical stability. In an effort to reduce the length of hospitalization for these patients, our institution has transitioned from inpatient (IP) to outpatient (OP) administration of BEAM conditioning. Here, we report the results of an analysis of the feasibility, cost, complications, and outcomes for the initial group of patients who received OP BEAM compared to a prior cohort of patients who received IP BEAM. Patient and disease characteristics were comparable for the two cohorts, as were engraftment kinetics. Length of hospital stay was reduced by 6 days for the OP cohort (*P *<* *0.001), resulting in a cost savings of more than $17,000 per patient. Fewer complications occurred in the OP cohort, including severe enteritis (*P *=* *0.01), organ toxicities (*P *=* *0.01), and infections (*P *=* *0.04). Overall survival rate up to 3 years posttransplant was better for the OP cohort (*P *=* *0.02), likely due to differences in posttransplant therapies. We conclude that OP administration of BEAM conditioning is safe and may offer significant advantages, including decreased length of hospitalization, reduced costs, decreased risks for severe toxicities and infectious complications, and likely improvement in patient satisfaction and quality of life.

## Introduction

High‐dose chemotherapy followed by autologous hematopoietic stem cell transplantation (ASCT) is standard consolidative treatment for patients with Hodgkin (HL) or non‐Hodgkin lymphoma (NHL) that persists or recurs following standard chemotherapy and/or radiation 1, 2, 3, 4, 5. The BEAM regimen (BCNU, etoposide [ETP], Ara‐C, and melphalan) is often utilized as the conditioning regimen and has traditionally been administered over 6 days in the hospital, with patients remaining hospitalized until hematologic recovery and clinical stability 6, 7, 8, 9. However, there is currently substantial interest in performing various aspects of stem cell transplantation (SCT) in outpatient (OP) settings and reports from multiple institutions suggest this can be done safely 10, 11, 12, 13. The feasibility of this approach, however, may depend on the facilities, infrastructure, and staffing of the institution, as well as the demographics of the patient population served 14, 15. The blood and marrow transplant (BMT) program at the JP Wilmot Cancer Institute (JPWCI) is the only comprehensive BMT program serving central New York State, a largely rural area of about 25 counties that comprises about half the square mileage of NY. In 2011, in an effort to reduce the length of hospitalization for patients undergoing ASCT for lymphoma, we transitioned from inpatient (IP) to OP administration of BEAM conditioning. We anticipated that this approach would not only result in significant cost savings and more efficient hospital bed utilization, but could also improve patient satisfaction and quality of life. In order to evaluate the impact of this approach at the JPWCI, we retrospectively evaluated the first 58 patients receiving OP BEAM and a comparable cohort of patients who received IP BEAM in the time immediately preceding the transition. This study describes the comparison between these two cohorts with respect to engraftment, length of hospital stay, toxicities, infectious complications, survival, and cost.

## Patients and Methods

### Patients

Following Institutional Research Subjects Review Board approval, we performed a retrospective chart review on all 58 patients who received OP BEAM conditioning prior to ASCT for lymphoma from January 2011 through March 2014. There were no specific exclusions, although four patients enrolled on a clinical trial specifying administration of ETP and Ara‐C every 12 h were admitted to the hospital and thus excluded. We also reviewed charts of the 49 consecutive patients who received IP BEAM conditioning immediately prior to the transition to OP BEAM. These patients underwent ASCT from July 2008 through December 2010. In addition, 31 patients received IP BEAM during the concurrent OP BEAM time period due to lack of a caregiver or physician preference, these patients were reviewed separately.

### Conditioning regimens

Patients receiving IP BEAM were admitted the first day of conditioning, while those receiving OP BEAM were admitted prior to the stem cell infusion on day 0. All patients remained hospitalized until hematologic recovery and resolution of active medical issues.

Patients receiving OP BEAM were required to stay within 30 min of the JPWCI and have a full‐time caregiver. Both patient and caregiver had to demonstrate an adequate level of comprehension regarding treatment plans and potential complications. Patients were evaluated by a nurse practitioner and an attending physician on the first day of conditioning and daily thereafter by either or both. During those visits, patients were directly queried about nausea, vomiting, diarrhea, infectious symptoms, and other issues, and were given a phone number for the SCT nurse practitioner should they have any problems after clinic hours. Laboratory monitoring included daily CBCs and chemistries.

**Figure 1:**
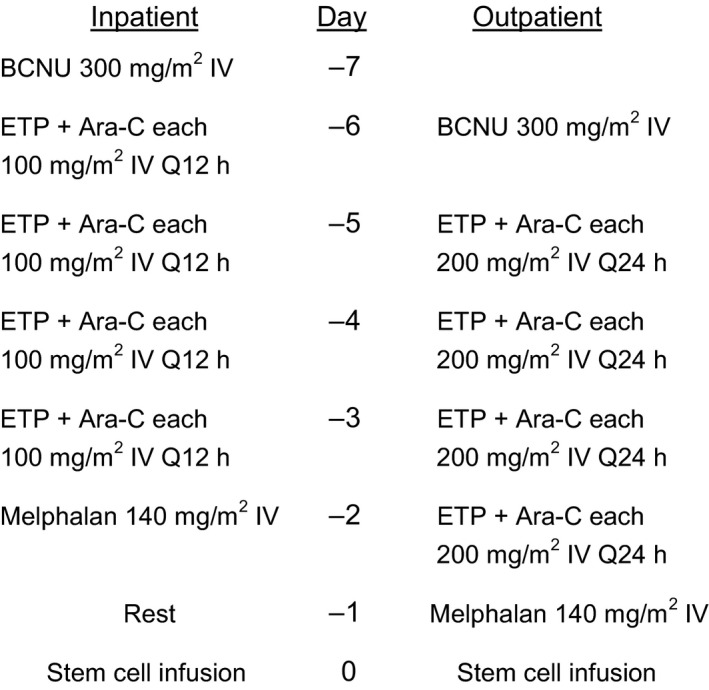
Inpatient and outpatient BEAM‐conditioning regimens. BCNU: carmustine; ETP: etoposide; Ara‐C: cytarabine. Chemotherapy drugs were dosed on 25% corrected ideal body weight: CIBW = IBW + [(0.25) × (Actual BW – IBW)], where IBW in kg = 50 (male) or 45.5 (female) + (2.3 × height in inches over 60 inches).

The IP and OP BEAM regimens utilized the same chemotherapeutic agents at the same total doses, but on slightly different schedules, as depicted in Figure 1. The modifications for OP BEAM chemotherapy were initially made to facilitate outpatient administration; however, this has since become our standard regimen for IP BEAM as well.

### Supportive care

Institutional guidelines for blood product support and symptom management were followed. Fluoroquinolone prophylaxis was started when the ANC dropped below 500/*μ*L. Broad‐spectrum antibiotics were started for fevers and continued until neutrophil recovery or the completion of specific therapy if a pathogen was isolated. Viral prophylaxis with (val)acyclovir was started with conditioning and continued for 6 months posttransplant. Fluconazole for fungal prophylaxis was started with conditioning and given until engraftment.

### Methods and definitions

Patients undergoing ASCT for lymphoma were identified by searching the Microsoft Access database used at our institution to track all patients undergoing SCT. Electronic medical records for each patient were then reviewed to confirm baseline demographic data and extract additional information. Pretransplant data acquired included specific diagnosis, disease status, lines of therapy received prior to transplant and response to each therapy, and comorbidity index as calculated by the Hematopoietic Cell Transplant‐Specific Comorbidity Index (HCT‐CI) 16. All patients underwent disease‐specific restaging studies prior to transplantation. A line of prior therapy was defined as a specific chemotherapy regimen or course of radiation prior to, but not including, conditioning.

All patients received mobilized peripheral blood stem cells. Times to neutrophil and platelet engraftment followed CIBMTR guidelines: for neutrophils, the first of ≥3 consecutive days with ANC ≥500/*μ*L without growth factor support; for platelets, the first of ≥3 consecutive days with platelet count ≥20,000/*μ*L without transfusion or, if the patient was transfused, 7 days following transfusion (if platelets remained ≥20,000). The National Cancer Institute Common Terminology Criteria for Adverse Events (NCI‐CTCAE), version 4.03, was used to report toxicities and infections. Diagnosis of infection required a positive culture, molecular pathogen identification, or radiographic evidence of disease in the setting of corresponding signs and/or symptoms and the need for systemic antimicrobial therapy.

Costs for OP and IP care were determined by review of financial records for lymphoma patients undergoing BEAM conditioning and ASCT. Average daily costs during OP BEAM and IP care were used to estimate cost savings for OP BEAM.

### Statistics

Continuous variables were compared using the nonparametric Wilcoxon test, and were summarized by the median, minimum, and maximum. Categorical variables were compared using Fisher's exact test and were summarized by counts and proportions. Infection densities were compared using the exact binomial test. Overall survival (OS) was defined as the time from ASCT to the date of death from any cause and progression‐free survival (PFS) as the time from ASCT to date of relapse, progression, or death, whichever occurred first. The Kaplan–Meier method was used to estimate the distributions of OS and PFS. The log‐rank test was used to assess differences in survival between the cohorts. Hypothesis tests were two‐sided and conducted at the 0.05 level of significance. All analyses were conducted using SAS version 9.4 (SAS Institute, Inc.).

## Results

### Patient and disease characteristics

As shown in Table 1, patient demographics, specific diagnosis, disease status, prior therapy, and HCT‐CI were comparable for the OP and IP BEAM cohorts. Median follow‐up was slightly longer for the IP cohort (36.9 vs. 31.4 months).

**Table 1:** Patient, disease, and treatment characteristics

* *	IP BEAM (*N* = 49)	OP BEAM (*N* = 58)	*P* value
Age (years)			0.83
Median	59	58	
Range	16–74	17–72	
Gender			0.69
Male	30 (61%)	38 (66%)	
Female	19 (39%)	20 (35%)	
Diagnosis	** **	** **	0.92
Follicular	4 (8%)	4 (7%)	
FL to DLBCL	4 (8%)	9 (16%)	
DLBCL	11 (23%)	15 (26%)	
Mantle cell	14 (29%)	15 (26%)	
Marginal zone	1 (2%)	1 (2%)	
Hodgkin	11 (22%)	9 (16%)	
Other	4 (8%)	5 (9%)	
Disease status			0.69
CR 1	14 (29%)	19 (33%)	
CR ≥2	15 (30%)	14 (24%)	
PR	19 (39%)	25 (43%)	
PD	1 (2%)	0	
Lines of prior therapy			0.23
1	11 (22%)	9 (16%)	
2	16 (33%)	28 (48%)	
3	14 (29%)	17 (29%)	
>3	8 (16%)	4 (7%)	
HCT‐CI			0.85
0	23 (47%)	31 (54%)	
1	9 (18%)	10 (17%)	
2	5 (10%)	5 (9%)	
3	8 (16%)	5 (9%)	
4	2 (4%)	3 (5%)	
>4	2 (4%)	4 (7%)	
Median follow‐up (months)	36.9	31.4	
Range	1.8–88.5	3.3–55.9	

IP, inpatient; OP, outpatient; FL, follicular lymphoma; DLBCL, diffuse large B‐cell lymphoma; CR, complete remission; PR, chemosensitive partial response; PD, persistent refractory disease; HCT‐CI, Hematopoietic Cell Transplant‐Specific Comorbidity Index.

### Cell dose, engraftment, and length of hospital stay

CD34^+^ cell doses and times to engraftment were comparable for IP and OP BEAM (Table 2). Length of hospitalization was reduced by 6 days with OP BEAM. No OP BEAM patient required early admission for toxicity, symptom management, infection, or patient/family request; one patient was admitted on day ‐2 to utilize an available inpatient bed.

**Table 2:** Transplant outcomes

	IP BEAM	OP BEAM	*P* value
CD34^+^ cell dose (×10^6^/kg)			
Median	4.18	4.59	0.72
Range	2–18.9	2–16.8	
Neutrophil engraftment (day)			
Median	10	10	0.33
Range	7–22	8–13	
Platelet engraftment (day)			
Median	10	11	0.17
Range	0–19	7–35	
Length of hospital stay (days)			
Median	18	12	<0.0001
Range	15–30	10–28	

IP, inpatient; OP, outpatient.

### Toxicity

Table 3 compares gastrointestinal (GI) and other organ toxicities experienced by patients receiving IP and OP BEAM. The JPWCI converted to an electronic medical record around the time of transition from IP to OP BEAM. Thus, details pertaining to GI or other toxicities were easier to quantitate for the OP BEAM cohort. Nonetheless, for the IP cohort, detailed discharge summaries that included stool volumes and documentation of other toxicities, as well as all laboratory, microbial, and radiographic information were available. Of note, with the exception of nausea/vomiting, no GI or other organ toxicities were recorded in either group prior to day 0.

**Table 3:** Transplant‐related toxicities

	IP BEAMNumber of patients (%)	OP BEAMNumber of patients (%)	*P* value

Nausea/Vomiting			0.29
Grades 0–1	32 (65%)	44 (76%)	
Grades 2–3	17 (35%)	14 (24%)	
Mucositis			0.46
Grades 0–1	38 (78%)	49 (85%)	
Grades 2–3	11 (22%)	9 (16%)	
Diarrhea/Enteritis	overall *P*‐value		0.004
Grades 0–1	25 (51%)	43 (74%)	0.02
Grades 2–3	18 (37%)	15 (26%)	0.29
Grade 4	6 (12%)	0 (0%)	0.01
Organ toxicity ≥ Grade 2			
Number of patients	14 (29%)	5 (9%)	0.01
Number of toxicities	21	6	
Grade 2	9	2	
Grade 3	9	4	
Grade 4	3	0	
Number of toxicities/patient			0.03
0	35	53	
1	9	4	
2	3	1	
3	2	0	
Types of organ toxicity (events)			
Afib	4	2	
Cardiac	5	1	
Pulmonary	4	1	
Renal	3	1	
Hepatic	4	0	
CNS	1	1	
MICU transfer	2 (3%)	0	

IP, inpatient; OP, outpatient; Afib, atrial fibrillation; CNS, central nervous system; MICU, medical intensive care unit.

While the incidences of grades 2–3 nausea/vomiting and mucositis were comparable for IP and OP BEAM, the IP cohort experienced almost twice the incidence of severe enteritis. Furthermore, no OP BEAM patient developed grade 4 enteritis, whereas six IP BEAM patients suffered this degree of toxicity.

Compared to OP BEAM, the IP cohort also had a significantly higher incidence of non‐GI organ toxicities, more severe organ toxicities, and more patients with multiorgan toxicities. Organ toxicities grade ≥2 are summarized in Table 3. There was no transplant‐related mortality for either cohort.

Shortly after implementation of OP BEAM, we changed our standard IP BEAM regimen to correspond to the OP regimen. In order to assess whether daily dosing of ETP and Ara‐C might result in less toxicity, we also reviewed charts on the patients receiving this BEAM regimen as inpatients during the same time period as our OP BEAM cohort. While the number of patients was low (*N *=* *17), the toxicities appeared similar to the prior IP BEAM cohort; one patient had grade 4 enteritis and there were 10 organ toxicities ≥grade 2.

### Infections

As shown in Table 4, the incidence of neutropenic fever was similar for OP and IP BEAM. However, the incidence of infection (density) was significantly higher for the IP cohort (*P *=* *0.04). One patient in the IP cohort developed varicella zoster on day ‐5; there were no other infections prior to day 0 in either cohort. The majority of infections were caused by common bacteria, with respiratory viruses being next most common. There was one case each of Mycobacterium avium, varicella zoster, hepatitis C reactivation, Streptomyces, and mold, all in the IP cohort.

**Table 4:** Infections within 30 days of transplant

	IP BEAM	OP BEAM	*P* value
Number of patients (%)			
Neutropenic fever	33 (67%)	30 (52%)	0.12
Infection	22 (45%)	15 (26%)	0.04
Incidence of infection (density)^a^	1.90	0.98	0.04
Number of infections			
All infections	28	17	
Types of infection			
Bacteremia	4	3	
Pneumonia	4	5	
UTI	3	3	
C. diff	5	5	
Other GI	6	1	
Cellulitis	4	0	
Other	2	0	

IP, inpatient; OP, outpatient; UTI, urinary tract infection; C. diff, *Clostridium* difficile colitis; GI, gastrointestinal.

Other GI: typhlitis, toxic megacolon, diverticulitis; other infections: hepatitis C, varicella zoster virus.

a Incidence of infection (density) = Number of infections/patient days × 100, where days = 30.

### Survival and posttransplant therapies

**Figure 2:**
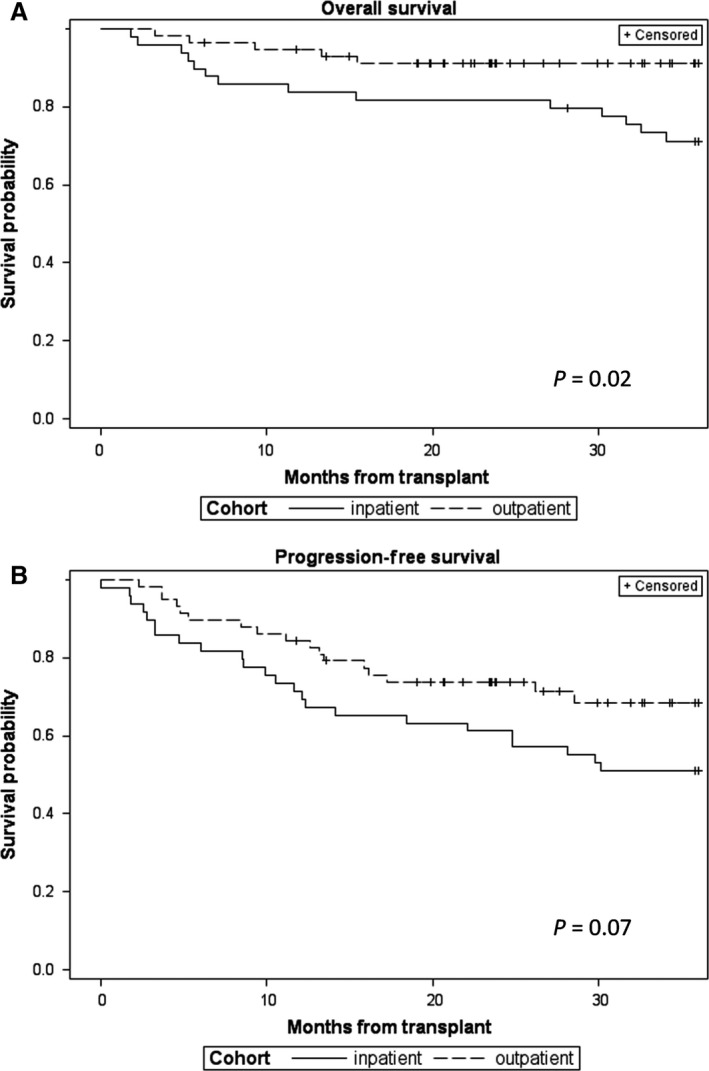
Kaplan–Meir plots demonstrating overall survival (A) and progression‐free survival (B) for inpatient and outpatient BEAM cohorts.

**Figure 3:**
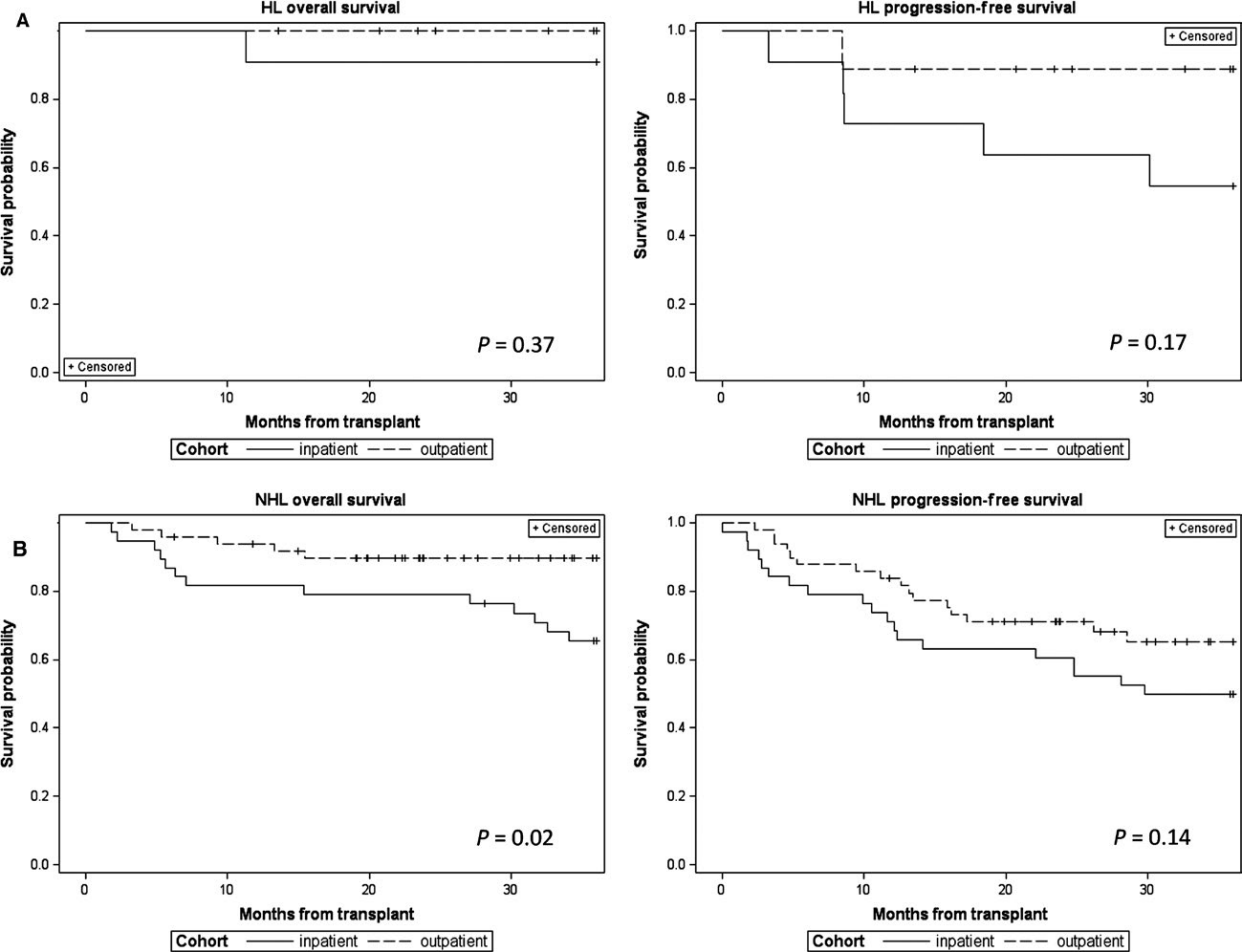
Kaplan–Meir plots demonstrating overall survival and progression‐free survival for Hodgkin lymphoma (A) and non‐Hodgkin lymphoma (B) subsets of the inpatient and outpatient BEAM cohorts.

As secondary analyses, we reviewed time to relapse or progression, survival, and cause of death for all patients. As illustrated in Figure 2, the OS rate up to 3 years posttransplant appeared to be better for the OP than the IP BEAM cohort (*P *=* *0.02) and there was a trend toward improved PFS in the OP cohort (*P *=* *0.07). Speculating that differences in posttransplant therapies for the two cohorts might be responsible for the differences in survival, we evaluated posttransplant therapies for all patients (Table 5) and analyzed OS and PFS for HL and NHL subsets of IP and OP BEAM (Fig. 3). Analyses of the HL subsets suggested improved PFS for the OP BEAM cohort, but the differences were not statistically significant, likely due to the small numbers of patients. Analyses of the NHL subsets demonstrated a significant difference in OS (*P *=* *0.02) and a trend toward improved PFS for the OP BEAM cohort.

**Table 5:** Posttransplant therapies

A. Therapies prior to relapse/progression			
	IP BEAM (*N* = 49)	OP BEAM (*N* = 58)	*P* value
Number of patients receiving:			
Consolidative radiation	3	10	0.14
Rituximab maintenance	2	3	
Brentuximab vs placebo	1	2	
Intrathecal chemotherapy	1	0	
	Overall *P* value		0.33

B. Initial therapy for relapse/progression occurring less than 18 months post‐ASCT			
	IP BEAM (*N* = 16)	OP BEAM (*N* = 15)	*P* value
Number of patients receiving:			
Chemotherapy	5	2	
Novel agent	4	11	0.01
Radiation	1	1	
No therapy	4	1	
Unknown	2	0	
	Overall *P* value		0.06
Number of patients receiving the following novel agents:			
Brentuximab	2	2	
Carfilzomib + Vorinostat	2	1	
Lenalidomide		2	
Bortezomib		1	
PI3K inhibitor + Jak1 inhibitor		2	
Amplimexon		1	
Ibrutinib		1	
Fostamatinib		1	

IP, inpatient; OP, outpatient; ASCT, autologous stem cell transplantation; PI3K, phosphoinositide 3‐kinase; Jak, janus kinase.

While the differences in consolidative (pre‐relapse) posttransplant therapies were not statistically significant, five of the nine OP HL patients and none of the 11 IP HL patients received consolidative radiation (Table 5A). Since all of the patients were at least 18 months posttransplant, we compared first‐line therapies to treat relapses occurring within 18 months of transplant (Table 5B). This analysis revealed chemotherapy or no therapy being used more often in the IP cohort and novel therapies predominating in the OP cohort.

### Cost

Excluding the cost of chemotherapy drugs, the cost of inpatient care for lymphoma patients undergoing BEAM and ASCT was an average of $3300 per day, while the average daily cost for outpatient BEAM administration was $400. Therefore, based on a decrease in hospital stay by 6 days, we estimated a cost savings of about $17,400 per patient for OP BEAM.

## Discussion

This cohort comparison between IP and OP administration of BEAM conditioning prior to ASCT for patients with lymphoma demonstrated that OP BEAM reduced the length of hospital stay and cost of treatment, and additionally was associated with lower rates of severe enteritis, organ toxicities, and infections. Although the study is limited by its retrospective nature, relatively small number of patients, and sequential time frames for the two cohorts, we conclude that at a minimum OP BEAM is feasible, safe, and cost‐effective.

Reports in the late 1990s first demonstrated the feasibility of OP ASCT for lymphoma 10, 17, 18. Outpatient care was generally restricted to patients having good performance scores and no significant comorbidities, and further to patients choosing to receive OP care 19. While those reports indicated that selected patients undergoing ASCT could be managed safely in an OP setting, the general applicability of the approach remained unclear and did not become widely utilized 20. Over the past decade, there has been resurgent interest in OP SCT and multiple centers have reported their experiences performing various aspects of ASCT in the OP setting 12, 13, 14, 21, 22, 23, 24, 25. While these reports are extremely variable in terms of patient selection and scope of outpatient management, there is a frequent emphasis on early posttransplant discharge 22, 23, 24, 25. In contrast, our model employs OP conditioning and IP posttransplant care, an approach that may offer certain advantages.

The BEAM regimen is generally well tolerated with regard to immediate side effects and none of our outpatients required early hospital admission for medical issues. We did not exclude patients based on age or comorbidity index and found OP BEAM to be safe, even for older patients and those with significant comorbidities. The current practice at our institution is for all lymphoma patients undergoing ASCT to receive BEAM conditioning in the OP department unless it is not possible to identify a suitable caregiver.

The decreased incidences of infections and organ toxicities we observed for OP BEAM are likely interrelated, as infections often predispose to organ dysfunction. It is also possible that the daily dosing of ETP and Ara‐C in OP BEAM resulted in less toxicity than the every 12 h schedule used for IP BEAM. However, the toxicity profile for the cohort of patients receiving the OP BEAM regimen as inpatients argues against this explanation. Of note, there was no evidence for reduced efficacy of the OP BEAM regimen. It also remains possible that the IP and OP cohorts differed in unmeasurable ways with respect to performance status, severity of comorbidities, or degree of prior toxicities from chemotherapy resulting in the IP cohort being more susceptible to complications.

Others have also reported reduced infection rates for SCT patients treated as outpatients. In a retrospective evaluation of 671 patients undergoing ASCT in IP and OP settings, McDiarmid and colleagues found significantly fewer infections in the OP cohort 21. Several factors that affect severity of illness, complication rates, and outcomes in hospitalized patients include nutritional status, degree of physical conditioning, sleep quality, and sense of emotional well‐being 26, 27. We speculate that these factors contributed to the differences in complications between our IP and OP BEAM cohorts. By staying active, eating and sleeping well, and remaining socially engaged during the 6 days of BEAM conditioning, the outpatients may have been physically and psychologically more resilient during the posttransplant period of pancytopenia and mucositis/enteritis.

Infections and organ toxicities are responsible for significant morbidity and mortality associated with SCT and a decrease in these complications could impact SCT outcomes. In addition, the same physical and psychological factors that reduce complications may improve SCT outcomes. Thus, it is conceivable that OP conditioning contributed to the improved survival rates we observed for OP BEAM. However, given the sequential rather than concurrent time frames for the IP and OP cohorts, we presume the better survival rates for OP BEAM primarily reflect improved therapies for lymphoma in the more recent time period. In particular, the increased availability of novel, more directed therapies in recent years likely improved the OS of these patients. While the explanation for the trend toward improved PFS is less clear, it seems probable that improved therapies also impacted relapse rates following ASCT. For example, we suspect that posttransplant consolidative radiation and/or brentuximab for HL patients reduced the risk of relapse for this group, although the number of patients in our study was too small to demonstrate significant differences for these factors. It is also possible that the degree of disease control at the time of transplant was better for the more recent OP cohort even though we did not find significant differences in disease status or lines of prior therapy between the IP and OP cohorts.

There are obvious advantages to OP SCT, but there are also some potential drawbacks, and a single model is unlikely to fit every institution. Factors to consider include patient demographics, caregiver and housing support, and infrastructure and resources of the institution 15, 20. A variety of OP SCT models have been described by different institutions, most of which are based on early discharge posttransplant. While there is a consensus that OP approaches are generally safe and cost‐effective, the feasibility and cost savings vary greatly 13, 17, 24, 28, 29, 30. For many institutions, providing extensive OP services for complex patients during an unpredictable pancytopenic period may abrogate many of the benefits of OP SCT. For example, in a randomized study of early discharge post‐ASCT, Faucher and colleagues found that only 40% of patients were actually discharged early and many were readmitted, resulting in a 1‐day difference in hospital stay and a 6% cost savings 23.

Outpatient BEAM has been extremely successful at our institution and has led to the implementation of several other OP‐conditioning regimens. This model of OP conditioning followed by IP posttransplant care facilitates planning for admissions, minimizes the need for additional personnel and resources, and optimizes cost savings. While the direct savings on cost of care for OP compared to IP BEAM is substantial, perhaps—from a global perspective—an even greater savings results from the reduction in resources consumed and the potential for more efficient hospital bed utilization. Most importantly, this general strategy is good for the overall medical and psychological care of these patients. In the future, as health care models continue to evolve, we need to be vigilant to assure that outpatient management of SCT patients remains excellent, that it improves the quality of life for patients and caregivers, and that cost savings for institutions are not simply shifted onto patients.

## Conflict of Interest

None declared.
